# Cortisol promotes breast‐to‐brain metastasis through the blood‐cerebrospinal fluid barrier

**DOI:** 10.1002/cnr2.1351

**Published:** 2021-02-26

**Authors:** Robert A. Herrera, Krutika Deshpande, Vahan Martirosian, Behnaz Saatian, Alex Julian, Rachel Eisenbarth, Diganta Das, Mukund Iyer, Josh Neman

**Affiliations:** ^1^ Department of Molecular Microbiology and Immunology Keck School of Medicine, University of Southern California Los Angeles California USA; ^2^ Department of Neurological Surgery Keck School of Medicine, University of Southern California Los Angeles California USA; ^3^ Department of Physiology and Neuroscience Keck School of Medicine, University of Southern California Los Angeles California USA; ^4^ Norris Comprehensive Cancer Center Keck School of Medicine, University of Southern California Los Angeles California USA; ^5^ Brain Tumor Center Keck School of Medicine, University of Southern California Los Angeles California USA

**Keywords:** BBB, BCSFB, breast‐to‐brain metastasis, cortisol

## Abstract

**Background:**

Elevated basal cortisol levels are present in women with primary and metastatic breast cancer. Although cortisol's potential role in breast‐to‐brain metastasis has yet to be sufficiently studied, prior evidence indicates that it functions as a double‐edged sword—cortisol induces breast cancer metastasis in vivo, but strengthens the blood‐brain‐barrier (BBB) to protect the brain from microbes and peripheral immune cells.

**Aims:**

In this study, we provide a novel examination on whether cortisol's role in tumor invasiveness eclipses its supporting role in strengthening the CNS barriers. We expanded our study to include the blood‐cerebrospinal fluid barrier (BCSFB), an underexamined site of tumor entry.

**Methods and Results:**

Utilizing in vitro BBB and BCSFB models to measure barrier strength in the presence of hydrocortisone (HC). We established that lung tumor cells migrate through both CNS barriers equally while breast tumors cells preferentially migrate through the BCSFB. Furthermore, HC treatment increased breast‐to‐brain metastases (BBM) but not primary breast tumor migratory capacity. When examining the transmigration of breast cancer cells across the BCSFB, we demonstrate that HC induces increased traversal of BBM but not primary breast cancer. We provide evidence that HC increases tightness of the BCSFB akin to the BBB by upregulating claudin‐5, a tight junction protein formerly acknowledged as exclusive to the BBB.

**Conclusion:**

Our findings indicate, for the first time that increased cortisol levels facilitate breast‐to‐brain metastasis through the BCSFB—a vulnerable point of entry which has been typically overlooked in brain metastasis. Our study suggests cortisol plays a pro‐metastatic role in breast‐to‐brain metastasis and thus caution is needed when using glucocorticoids to treat breast cancer patients.

## 1 INTRODUCTION

Metastatic brain tumors bear a dismal 6‐month median survival time, constituting greater than 90% of all brain tumors.^1^ The most common primary tumors sites are lung (50%), followed by breast (20%) which together comprise roughly two‐thirds of cases. Unfortunately, despite the high frequency of metastatic brain tumors, there remains no universally accepted paradigm for chemotherapy treatment.^2^ Conventional chemotherapy has historically played a muted role in brain metastasis management due to inherent barrier properties of the central nervous system (CNS).^3^ Furthermore, we and others have shown that the brain's microenvironment has a promoting effect on metastatic tumors.^4^, ^5^, ^6^, ^7^, ^8^, ^9^, ^10^, ^11^, ^12^

The CNS is tightly separated from the dynamic milieu of blood by the blood–brain‐barrier (BBB) and the blood‐cerebrospinal fluid barrier (BCSFB). The BBB is a highly specialized structure connected by the interactions of vascular brain endothelial cells (BECs), an ensheathed basement membrane housing pericytes, and projecting astrocytic foot processes. Unlike peripheral endothelial cells, BECs are nonfenestrated and inhibit the untethered paracellular diffusion of water‐soluble molecules by an interconnected network of tight junctions (TJs).^13^, ^14^ Analogous to the endothelial barrier, the BCSFB correlate are apical TJs expressed within choroid plexus epithelial cells (CPEs) preventing the free flow of water‐soluble molecules. These choroid plexus cells have a supplemental secretory function—production of cerebrospinal fluid (CSF) which extravasates into the brain's ventricles and is dispersed throughout the CNS.

Tumor colonization into the brain parenchyma requires successful breaching of these CNS barriers. Prior in vitro transendothelial/epithelial electrical resistance (TEER) assays have demonstrated that the BBB (80‐100 Ω × cm^2^) is stronger and less permeable than the BCSFB (30‐40 Ω × cm^2^).^15^, ^16^ This discrepancy can be attributed to their roles in CNS homeostasis regulation; the BBB's primary function is to inhibit all paracellular diffusion to protect the brain from invading pathogens whereas the BCSFB requires increased permeability to support water intake into the CSF.^17^, ^18^

The BBB and BCSFB differ in their TJ architecture, employing different occludin, claudin, and junctional adhesion molecules.^19^ Claudin‐5 is the most abundant and primary TJ protein for barrier formation in the BBB whereas the BCSFB correlate is claudin‐1 and claudin‐3.^17^, ^20^, ^21^ Recently, expression of claudin‐5 in CPEs was observed which previously was reported to be specific to BBB.^22^

Cortisol, a glucocorticoid steroid hormone, targets BBB endothelial cells and upregulates claudin‐5 expression generating a tighter barrier.^23^ Interestingly, elevated basal cortisol levels are present in women with early stage breast cancer (0.49 μmol/L) and metastatic breast cancers (0.70 μmol/L) when compared to healthy women (0.29 μmol/L). However, cortisol exhibits a stark duality—glucocorticoids such as dexamethasone induce breast cancer metastasis in vivo,^24^ albeit with no mention to brain metastasis.

Our study determines whether elevated levels of cortisol, induced by tumor formation, will effect brain metastasis by reinforcing the tight junctions of the BBB and BCSFB.

## 2 MATERIALS AND METHODS

### 2.1 Cell maintenance and growth

Human cerebral microvessel endothelial cells (HCMEC), human cerebral vascular pericytes (HCVP), and human astrocytes were purchased from ScienCell Research Laboratories (Carlsbad, California) and used for BBB formation in vitro. BBB cells were cultured separately in Artificial BBB medium containing 50% Advance DMEM/F12 (Gibco Life Technologies, Waltham, Massachusetts), 50% neurobasal‐A medium (Gibco Life Technologies, Waltham, Massachusetts), 1% Anti‐Anti (Gibco Life Technologies, Waltham, Massachusetts), 1% GlutaMAX (100×) (Gibco Life Technologies, Waltham, Massachusetts), 5% fetal bovine serum (Omega Scientific, Tarzana, California), and 1% B‐27 supplement (50×) (Thermo Fisher Scientific, Waltham, Massachusetts). The cell line used for the blood‐cerebral‐spinal‐fluid‐barrier (BCSFB) was human choroid plexus epithelial (CPE) cells (ScienCell Research Laboratories, Carlsbad, California). The tumor cell lines used were lung adenocarcinoma (A549), breast cancer (MDA‐MB231), patient‐derived lung to brain metastasis (LuBM5), and patient‐derived breast to brain metastasis (BBM3.1) obtained from neurological surgery resections at USC with appropriate patient consent. Tumor cells and CPE cells were cultured in advanced DMEM/F12, supplemented with 1% Anti‐Anti, 1% GlutaMAX, and 10% FBS. Tumor cells and HCMECs were grown on plastic while CPEs, astrocytes, and pericytes were grown on collagen I, rat tail (Thermo Fisher Scientific, Waltham, Massachusetts) coated flasks. All cell lines were grown and maintained in a humidified incubator at 37°C and 5% CO_2_. All experiments were performed in triplicates, all data are expressed as the mean ± SD. All cell lines have been authenticated using STR profiling and that all experiments were performed with mycoplasma‐free cells should be included. All patient‐derived cell lines were obtained through approved University of Southern California Institutional Review Board consent protocol.

### 2.2 BBB and BCSFB in vitro model

Twelve‐well plate transwell inserts (665 635, Greiner Bio‐One, Monroe, North Carolina) with 8.0‐μm^2^ pores were used to measure barrier integrity of the BBB and BCSFB in vitro. The transwell inserts were inverted and placed into a six‐well plate where the exterior side of the transwell was coated with rat tail collagen I. For BBB assays, the goal was to culture HCMECs on top of the transwell with astrocyte pericyte mixture on the bottom. In order to achieve this, 1:1 ratio of 100 000 astrocytes and pericytes were seeded onto the exterior side of the inverted transwells. After 24 hours, transwells were reinverted and 150 000 HCMECs in 1 mL of medium were seeded into the top chamber. BBB models were used 5 days after this setup. For BCSFB assays, 80 000 CPE cells were seeded similarly onto inverted transwells. Cells were seeded with 300 μL of their appropriate culture medium. Lids were placed onto the six‐well plates making contact with the 300 μL of medium on the inverted transwells which prevents evaporation. The cells were allowed to settle and adhere to the transwells overnight. The following day the transwells were reinverted to their normal positioning and placed into a 12‐well plate containing 1 mL of their appropriate medium. A total of 1 mL of medium without cells was placed into the top chamber of the BCSFB transwells. BCSFB models were used 5 days after this setup. Additionally, for co‐culture experiments with tumor cells, breast or lung cancer cells were seeded at 150000 cells on the bottom of transwell.

### 2.3 Treatments

After transwell cultures reached confluency, the medium was changed to a serum‐reduced barrier medium consisting of 0.25% FBS, DMEM, 1% Anti‐Anti, and 1% GlutMAX with or without 550 nM hydrocortisone (HC) (MilliporeSigma, Burlington, Massachusetts). After 48 hours of HC pretreatments, 15 μL of concentrated tumor conditioned medium was added to the top chamber of the transwell for a 24‐hour treatment with and without 550 nM HC if samples were pretreated with HC. The values for hydrocortisone treatment was used as reported by Forster et al.^23^

### 2.4 Concentrating conditioned medium

Tumor cells were seeded onto three wells of a six‐well plate at 150000 cells/well and 3 mL/well of their appropriate culture medium. The conditioned medium was collected after 48 hours. The collect was transferred to a 15‐mL conical tube and centrifuged at 2000 × *g* for 2 minutes. The supernatant was transferred to an Amicon Ultra‐15 Centrifugal Filter Unit (MilliporeSigma, Burlington, Massachusetts) where it was centrifuged at 5000 × *g* for 20 minutes. The filtrate was discarded and the retentate (~400 μL) was used for treatments or stored at –80°C for later use.

### 2.5 TEER assay

Transwell cultures were replenished with fresh medium after 2 days of initial seeding. The in vitro barrier TEER was measured in Ω × cm^2^ twice in a day according to the manufactures protocol until it reached the maximum resistance potential according to the manufacturers protocol of MilliCell ERS‐2 (MilliporeSigma, Burlington, Massachusetts). Confluency was observed when resistance peaked and leveled off (>40 Ω × cm^2^ for BCSFB, and >70 Ω × cm^2^ for BBB) which occurred after 4 days of initial seeding.

### 2.6 Permeability assay

Transwells with healthy BBB and BCSFB were washed twice with diffusion buffer (10 mM HEPES, 4.5% glucose, and 0.1% bovine serum albumin) (MilliporeSigma, Burlington, Massachusetts). A total of 900 μL diffusion buffer was placed in the top chamber (transwell insert) and 1 mL diffusion buffer placed into the bottom chamber. A total of 100 μL of 1 mg/mL sodium‐fluorescein (MilliporeSigma, Burlington, Massachusetts) was added to the top chamber and was allowed to diffuse to the bottom chamber (12‐well plate). After 30 minutes, the bottom chamber was gently mixed using a pipette, and 100 μL was removed and placed into a clear bottom 96 well plate. A total of 100 μL was removed again from the bottom chamber after an additional 30 minutes. The fluorescence (FL) of the 96‐well plate samples were measured using a FLUOstar Omega filer‐based multimode microplate reader (BMG Labtech, Cary, North Carolina) with absorbance at 460 and 515 nm, respectively. FL observed was directly proportional to permeability.

### 2.7 Tumor transmigration

BBB and BCSFB transwell cultures were set up as mentioned above on a 12‐well culture dish. Once the barriers have reached confluency and pretreated with or without 550 nM HC, a tumor migration gradient was formed by placing 1.5 mL of serum‐free media on the top chamber and 2.5 mL of 10% FBS media in the bottom chamber. (Tumor migration gradient will have 550 nM HC present, if sample was previously pretreated with HC). A total of 150 000 GFP positive tumor cells, previously cultured for 24 hours in serum‐free medium, were seeded into the top chamber of the transwell containing the tumor cell migration gradient. Tumor cells were allowed to migrate for 24 or 48 hours through the transwell mesh. A total of 500 μL of medium was replaced after 24 hours then incubated for another 24 hours. After incubation, transwell cultures were carefully removed. The tumor cells which failed to cross and adhered to the transwell wall were removed using a wet cotton swab. Later, the transwells were gently washed twice with 1× PBS and placed into a new 12‐well plate containing 1 mL of 1% paraformaldehyde and allowed to fix for 10 minutes. After fixation, the transwells were further washed twice in 1× PBS and allowed to air‐dry for 3 minutes. The transwell mesh was cut out of the insert using a razor and placed onto a microscope slide with the cells facing upward. A total of 10 μL of ProLong Gold Antifade Mountant with DAPI (Thermo Fisher Scientific, Waltham, Massachusetts) was added to the fixed sample, and a 22‐mm square cover glass was placed onto the sample. Clear nail polish was used to seal the cover glass. Seven fields from each mesh filter were imaged using a confocal microscope at 20× magnification. Images were acquired using only the GFP channel. GFP positive cells were counted and averaged among the seven fields. All fluorescent imaging was done in the Cell and Tissue Imaging Core of the USC Research Center for Liver Diseases.

### 2.8 Immunocytochemisty

To verify tight junction expression, CPE cells or HCEMCs were stained using the standard immunocytochemisty (ICC) immunofluorescence protocol. Cells were grown on 15 mm circular coverslips in a 24‐well plate and fixed in 4% PFA for 10 minutes. After two washes with PBS the cells were blocked using blocking buffer (50% seablock and 50% 1×—PBS) for 1 hour. The cells were then incubated overnight at 4°C with primary antibodies ZO‐1 (Thermo Fisher Scientific, Waltham, Massachusetts) and Claudin‐5 (Thermo Fisher Scientific, Waltham, Massachusetts) at of 1:200 and 1:100 dilutions, respectively. The following day, the cells were washed three times in 1×‐PBS for 5 minute per wash. Samples were then incubated with secondary antibodies cy3 (1:300) (Jackson ImmunoResearch Laboratories, West Grove, Pennsylvania) and AlexaFluor 488(1:300) (Jackson ImmunoResearch Laboratories, West Grove, Pennsylvania) for 1 hour combined with AlexaFluor 647 conjugated Phalloidin (1:200) (Thermo Fisher Scientific, Waltham, Massachusetts). Afterwards samples were washed three times for 5 minutes and mounted onto microscope slides containing ProLong Gold Antifade Mountant with DAPI. The coverslips were then sealed using nail polish and allowed to dry.

### 2.9 Quantitative PCR

Quantitative PCR was used to determine relative mRNA levels of target genes according to the manufacturers protocol using RNeasy Plus Mini (Bioline, Taunton, Massachusetts). Concentration and homogeneity of the isolated RNA was measured using a Nanodrop 8000 (Thermo Scientific, St. Louis, Missouri). The mRNA obtained was reverse transcribed to cDNA according to the prescribed protocol by Maxima First Strand cDNA Synthesis Kit (Thermo Scientific, St. Louis, Missouri) and was amplified using Vertiti 96 well Fast Thermal Cycler (Applied Biosystem, Waltham, Massachusetts). For amplification, two of cDNA was placed into a 96‐well PCR plate containing a mixture of 1 μL qPCR primers (Integrated DNA Technologies, Coralville, Iowa), 7 μL of nuclease free water, and 10 μL of SYBR Green Supermix (Bio‐Rad, Hercules, California). All samples were done in triplicates. The plates were then run using a StepOnePlus Real‐Time PCR System (Applied Bioststems, Waltham, Massachusetts). The results were analyzed using the C_T_ values and GraphPad Prism (GraphPad Software, San Diego, California).

## 3 RESULTS

### 3.1 Hydrocortisone and tumor conditioned medium influence barrier property of the BBB and BCSFB

**FIGURE 1:**
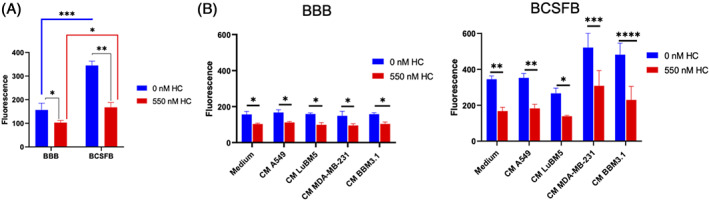
MDA‐MB‐231 and BBM 3.1 induces blood‐cerebrospinal fluid barrier (BCSFB) permeability in the presence of hydrocortisone (HC). Fluorescein permeability assay detects amount of sodium fluorescein that crosses the barrier. A, Comparison of the blood‐brain‐barrier (BBB) and BCSFB leakiness in the presence or absence of HC. B, Dose dependent effect of HC (0‐550 nM) on brain barriers with various conditioned medium (CM) cancer cell lines co‐culture. All experiments were performed in triplicates. Unpaired Student's *t* test (two tailed) was performed for two groups (0 nM HC and 550 nM HC). For multiple group analysis, one‐way analysis of variance (ANOVA) with Bonferroni tests was used. *P < .05, **P < .01, ***P < .001, ****P < .0001. ns, not significant

Hydrocortisone increases barrier tightness of the BBB,^23^ but its effect on the BCSFB remains to be investigated. Therefore, we now assess whether hydrocortisone can preserve the functional barrier integrity of the BBB and BCSFB in the presence of tumor conditioned medium. Our results show that at baseline, BCSFB was significantly (*P* < .001) more permeable than the BBB. Treatments with hydrocortisone showed decreased leakiness in both BBB and BCSFB. However, in the presence of HC the BCSFB was still significantly leakier (*P* < .05) than BBB (Figure 1A). Further, we examined leakiness of both barriers with tumor conditioned medium (CM). In the absence of HC treatment, the barrier performance of BBB co‐cultured with tumor cell conditioned medium was found to be preserved. However, a significantly compromised barrier function was observed with BCSFB when co‐cultured with CM MDA‐MB‐231 (*P* < .01) and CM BBM 3.1 (*P* < .05; Figure S1A). In contrast, BCSFB exhibited a minor change in permeability with CM A549 or CM LuBM5 (Figure S1A).

The in vitro brain barriers displayed significant reduction in permeability when treated with HC and various cancer cell condition media (CM; Figure 1B). Barrier function of the BBB treated with HC was stable in all tumor conditioned medium (Figure S1B). Similarly, BCSFB was able to retain its barrier property in co‐culture with CM A549 or CM LuBM5 medium, however, loss of barrier function was evident with CM MDA‐MB‐231 (308.05 ± 85.32 FL, *P* < .05) co‐culture compared to the control. Additionally, the HC‐treated BCSFB along with CM BBM3.1 showed a similar tendency of increasing permeability with no cogent difference (ns, *P* > .05) compared to the BBB (Figure S1B).

### 3.2 Breast cancer preferentially migrate through BCSFB over BBB

**FIGURE 2:**
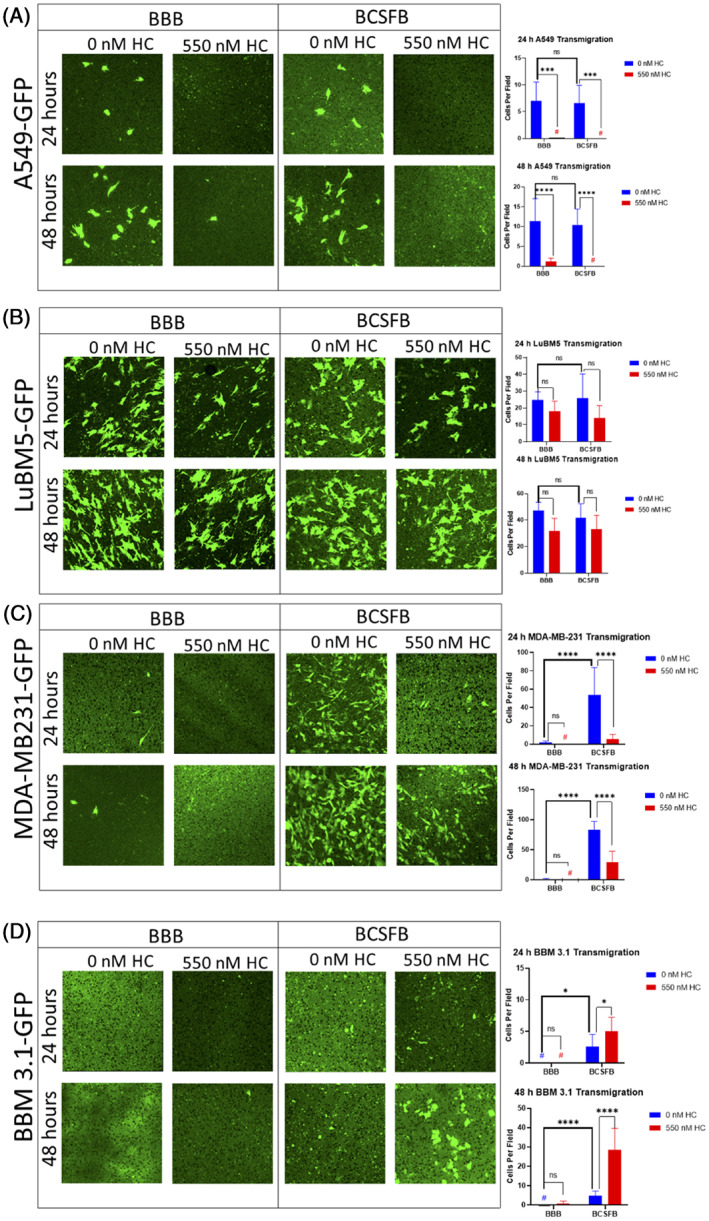
MDA‐MB‐231 and BBM 3.1 cells migrate efficiently through the blood‐cerebrospinal fluid barrier (BCSFB) than the blood‐brain‐barrier (BBB) in the presence of hydrocortisone (HC). A‐D, GFP positive tumor cell transmigration through BBB (left four panels) and BCSFB (right four panels) ±550 nM HC for 24 or 48 hours. A, A549‐GFP cells; B, LuBM5‐GFP cells; C, MDA‐MB‐231‐GFP cells; D, BBM 3.1 cells. Seven fields were captured, and cells were averaged among the fields. The unpaired Student's *t* test (two tailed) was used to measure variability. Images 40×. *P<0.05, **P<0.001, ***P<0.0001

We next assessed tumor cell migration across the BBB and BCSFB in a 24‐ and 48‐hour time dependent formats. First, we compared barrier properties of both the barriers co‐cultured with various cancer cell lines for 24 hours in presence and absence of HC. Our results show that in the absence of HC, migration of A549 (primary lung cancer) cells through BBB and BCSFB was not markedly different (Figure 2A). In the presence of HC, cell migration was completely inhibited at 24 hours (Figure 2A). After 48 hours, HC significantly (*P* < .0001) reduced migration of A549 cells across the BBB and also inhibited migration through the BCSFB (Figure 2A). LuBM5 cells showed nonpreferential migratory patterns similar to A549 cells in all conditions (Figure 2B).

Comparatively migration of MDA‐MB‐231 (*P* < .0001) through the BCSFB was observed whereas limited migration was recorded with the BBB at 24 hours in absence of HC (Figure 2C). However, treatment and incubation for 24 hours with HC inhibited MDA‐MB‐231 cell migration through the BBB while significantly reducing migration through the BCSFB. Further, we compared the barrier properties while treating with and without HC for 48 hours. In the absence of HC, migration of MDA‐MB‐231 through the BCSFB was significantly more (*P* < .0001) than the BBB. While MDA‐MB‐231 migration at 48 hours in the presence of HC was completely inhibited through the BBB, HC's constrictive barrier properties reduced cell migration through the BCSFB (*P* < .0001) relative to the control (Figure 2C); Similar results at 48 hours were also observed for the SKBr3 (Figure S2A). Interestingly, BBM 3.1 cells exhibited no migration through the BBB at 24 and 48 hours both with and without HC treatments. However, post 48 hours of HC treatments, BBM 3.1 showed a significant increase in cell migration through the BCSFB (Figure 2D); similar results at 48 hours were also observed for brain trophic MDA‐MB‐231Br (Figure S2B). Overall, these results show potential loss of BCSFB barrier properties in the presence of breast cancers.

### 3.3 Breast tumor environments degrade BCSFB tight junctions on CPE cells

**FIGURE 3:**
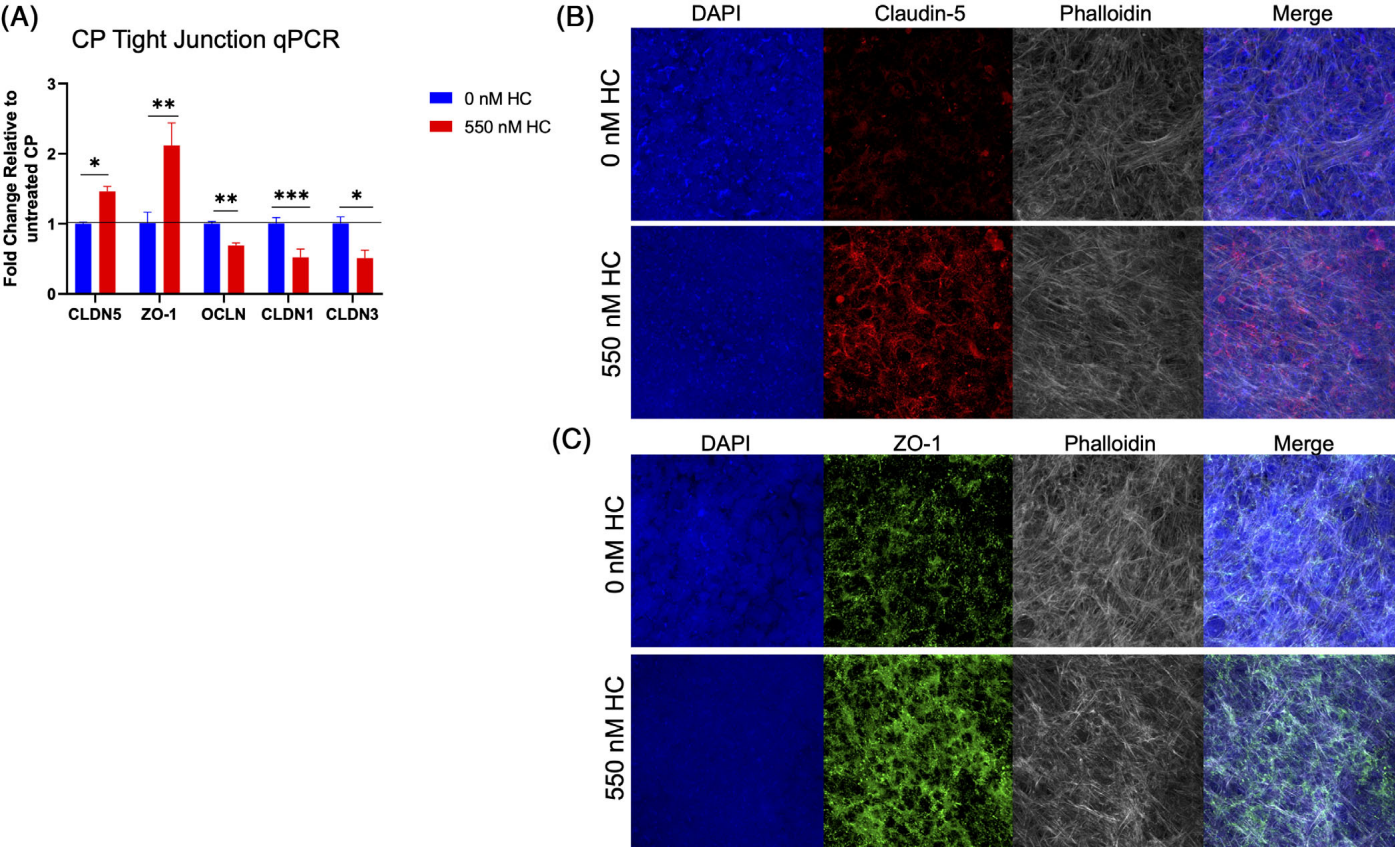
Hydrocortisone (HC) downregulates essential barrier‐forming tight junctions (TJs) in CP cells while upregulating CLDN‐5 and ZO‐1 on the mRNA and protein level. A, qPCR results. B,C, Claudin‐5, ZO‐1, Occludin, Claudin‐1, Claudin‐3 relative fold change to 0 nM HC. ICC of CP cells treated with 0 or 550 nM HC for 48 hours. B, DAPI blue, Claudin‐5 (red), Phalloidin (white), Merge. C, DAPI (blue), ZO‐1 (green), Phalloidin (white), merge. The unpaired Student's *t* test (two tailed) was used to detect statistically significant differences. *P < .05, **P < .01, ***P < .001, ****P < .0001. ns, not significant. Images 40×

**FIGURE 4:**
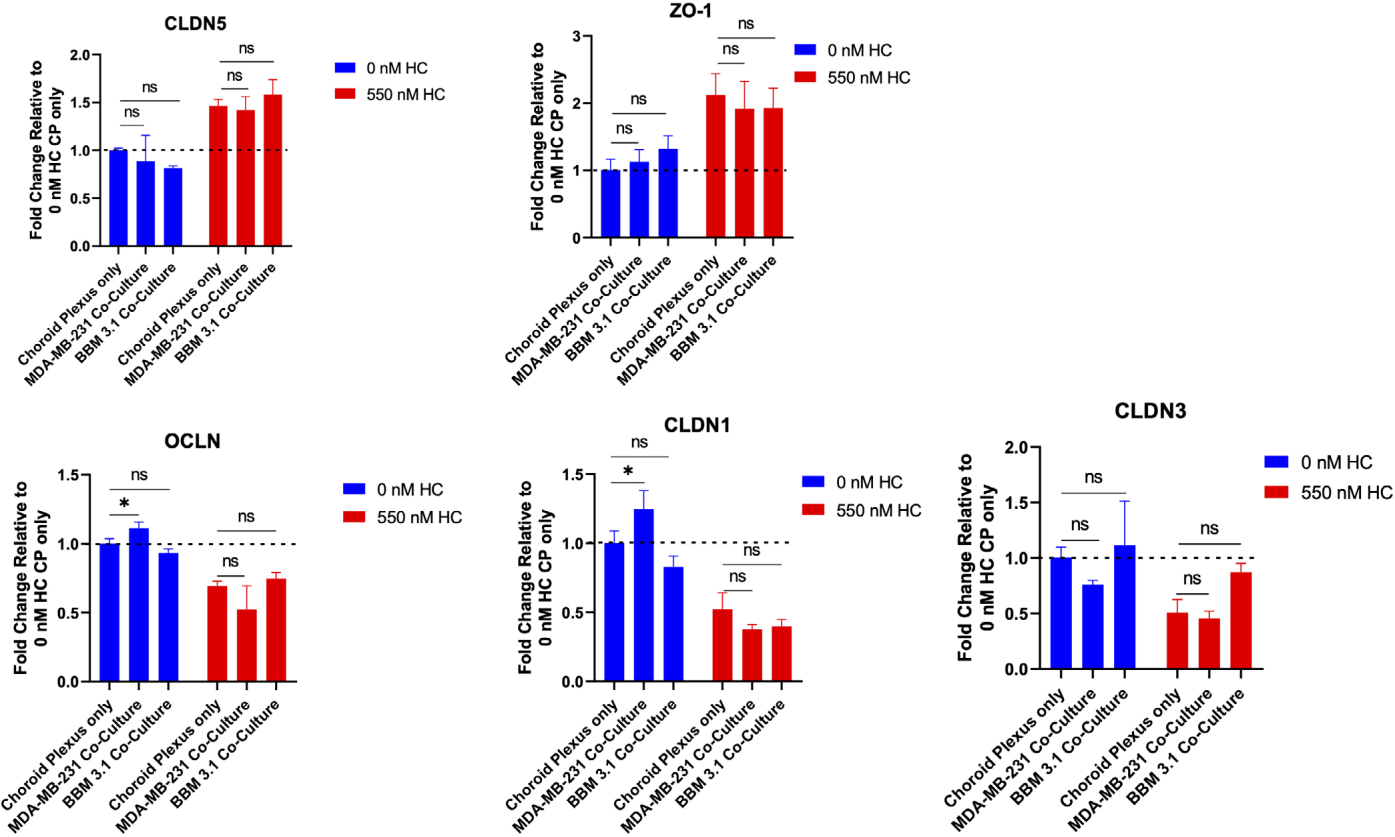
Breast tumor co‐cultures do not decrease tight junction gene expression. Bar graphs are fold changes relative to 0 nM hydrocortisone (HC) CP only. RPLPO gene expression was used to normalize data. For multiple group analysis, one‐way analysis of variance (ANOVA) with Bonferroni tests was used followed by statistical significance. *P < .05, **P < .01, ***P < .001, ****P < .0001. ns, not significant. Significance test was relative to CP only control within 0 nM HC and 550 nM HC

**FIGURE 5:**
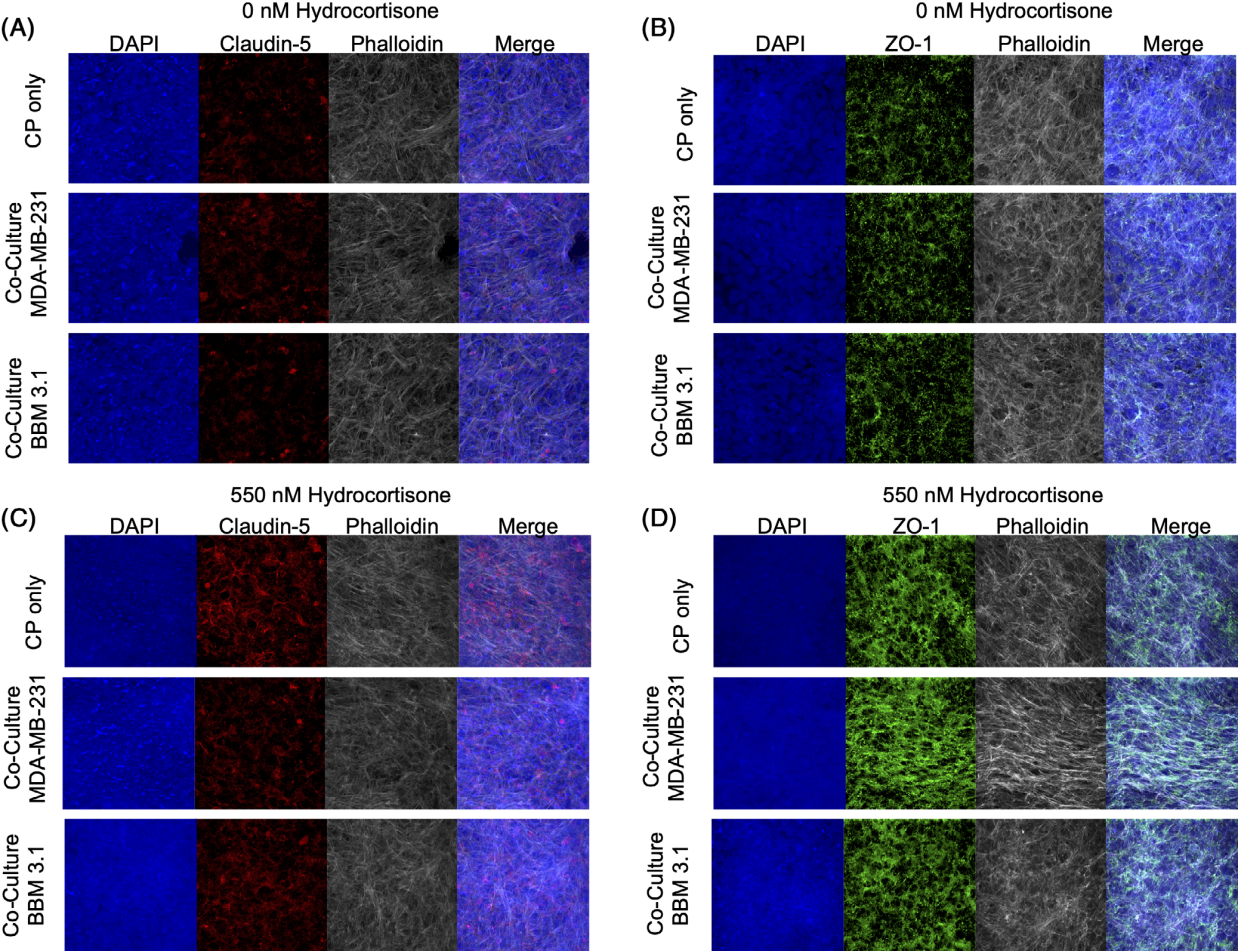
Breast tumor co‐cultures decrease claudin‐5 protein expression in the presence of hydrocortisone (HC). Fluorescent intensity threshold was determined by adjusting the fluorescent intensity until no signal was detected on negative primary antibody controls. These settings were used for imaging corresponding positive antibody samples (claudin‐5 or ZO‐1). All the treatments were for 48 hours. A,B, 0 nM HC, C,D, 550 nM HC. ICC stains includes DAPI (blue) Claudin‐5 (red), ZO‐1 (green), and Phalloidin (white). Images 40×

We next determined mRNA expression profiles of BCSFB tight junction proteins in tumor conditioned environment in the presence and absence of HC. qPCR results on CPE cells with HC treatment show increase of *Claudin‐5* (*P* < .01) and *ZO‐1 P* < .05) expression and significant downregulation of *Occludin* (*P* < .001), *Claudin‐1* (*P* < .05) and *Claudin‐3* (*P* < .05) (Figure 3A). The protein expression levels for Claudin‐5 and ZO‐1 were then confirmed (Figure 3B,C). Results show, similarly to mRNA levels, CPE ZO‐1 and Claudin‐5 have increased expression with HC treatment relative to controls. Previous permeability and transmigration assays demonstrated that BCSFB breakdown was susceptible to MDA‐MB‐231 and BBM 3.1 cells. Therefore, we evaluated mRNA expression profiles of various TJ proteins in presence and absence of HC co‐cultured with tumor cell lines. Results show there was a significant increase in *Occludin* and *Claudin‐*1 in CPE cultures relative to co‐cultures of CPE and tumor cells. However, when HC was added, there was no significant change in TJ proteins in CPE cultures relative to co‐cultures of CPE and tumor cells (Figure 4). Furthermore, in the absence of HC, Claudin‐5 and ZO‐1 protein expression remained consistent with tumor co‐cultures (Figure 5A,B). However, in the presence of HC, a decrease in Claudin‐5 expression was observed (Figure 5C). No change in ZO‐1 TJ protein across both the co‐cultures was visible (Figure 5D).

### 3.4 Hydrocortisone promotes tumor migration

**FIGURE 6:**
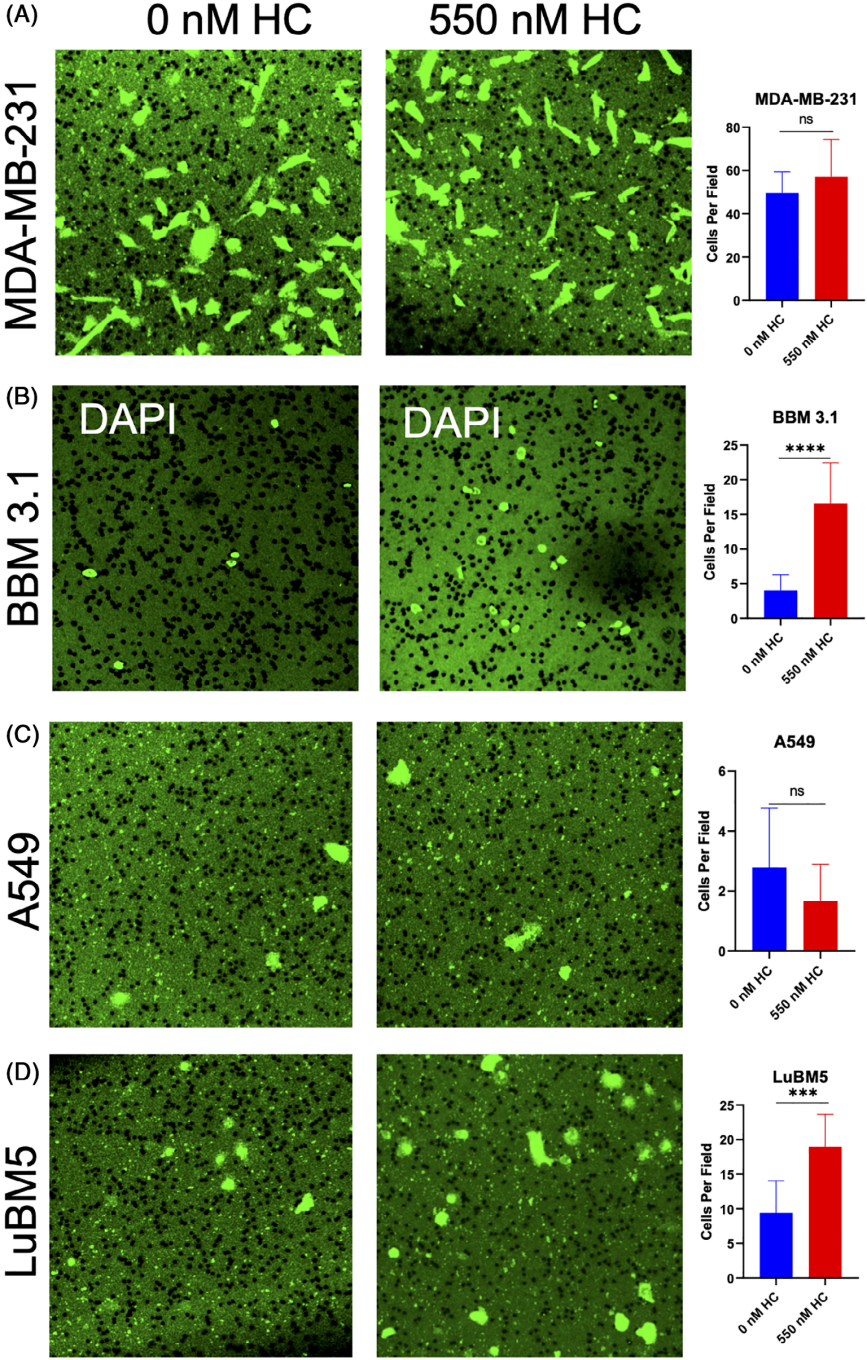
Hydrocortisone (HC) increases migration independent of barrier resistance in LuBM5 and BBM3.1. A‐D, GFP positive tumor transmigration through blank transwells ±550 nM HC for 12 hours. Graphs are quantifications of 0 nM HC vs 550 nM HC migrations, A, MDA‐MB‐231‐GFP, B, BBM 3.1 (DAPI was used since GFP signal was very low the first 12 hours of initial BBM 3.1 migration), C, A549‐GFP, and D, LuBM5‐GFP. Seven fields were captured for all the images and cells were averaged among the fields. The unpaired Student's *t* test (two tailed) was used to detect statistically significant differences. *P < .05, **P < .01, ***P < .001, ****P < .0001. ns, not significant. Images 40×

We analyzed whether HC has any effect on tumor migration independent of barrier resistance. MDA‐MB‐231 cells show no significant difference in migration rates between untreated and HC‐treated samples (Figure 6A). However, a significant increase in BBM 3.1 cell migration (*P* < .0001) was observed when treated with HC (Figure 6B). No difference was observed in A549 cell migration between untreated and HC‐treated samples (Figure 6C). Additionally, HC increased LuBM5 cell migration significantly (*P* < .001; Figure 6D). Overall, these results suggest HC induce tumor cell migration.

## 4 DISCUSSION

The glucocorticoid steroid, cortisol, increases in primary and metastatic breast cancer patients.^25^ Studies attempting to elucidate its mechanistic role have demonstrated a conflicting duality—cortisol promotes metastases systemically only in breast cancer, with no observation of brain metastasis .^24^ Additionally, the role of hydrocortisone was found to reinforces the blood‐brain‐barrier properties.^26^ However, no studies thus far have directly examined the role of cortisol on breast‐to‐brain metastasis. Our study furthers elucidates these inconsistencies by demonstrating that: (a) HC strengthens the BCSFB through increased Claudin‐5 and ZO‐1 expression; (b) breast cancer cells preferentially migrate through the BCSFB over the BBB; (c) HC increases the migratory nature independent of barrier resistance of metastatic breast cancer cells; and (d) HC increases metastatic breast cancer cell transmigration across the BCSFB. These results suggest cortisol's induction of metastatic breast cancer cell migration supersedes its ability to strengthen the BCSFB TJs, resulting in BCSFB traversal and potential brain colonization.

Prior studies have demonstrated that the BCSFB is weaker and more permeable than the BBB.^15^, ^16^ Consistent with these results, our study concomitantly shows that the BCSFB allows increased diffusion of fluorescein particles either in the absence or presence of HC. When examining this finding in the context of tumor conditioned media either with or without HC, the BCSFB remained leakier for only the breast cancer cells compared to lung cancers. We therefore propose that breast cancer cells secrete proteases into the extracellular environment that may degrade the BCSFB.

Our transmigration experiment furthers this notion, as breast cancer cells retained specificity toward the BCSFB over the BBB; while lung cancer cell lines lacked migration specificity and migrated across either barrier at comparable rates. If high levels of cortisol prevent breast tumor entry via BBB, then circulating breast tumor cells would continue to circulate through the BBB's vascular system until reaching a point of vulnerability such as the BCSFB. in vivo metastases experiments involving HC treatments and breast to brain localization would be needed to support this claim.

With increasing evidence for the BCSFB as a potential point of entry, our study determined HC's modulation of TJ protein expression on CPE cells in isolation. Our results demonstrate that HC treatment upregulates Claudin‐5 protein expression, while not effecting Claudin‐1 or ‐3 expression, which are regarded as the main barrier‐forming tight junctions of the BCSFB.^17^, ^20^, ^21^ Thus, our results suggests that exposure to high levels of cortisol induces the BCSFB to become more “BBB‐like” in order to protect the CNS from systemic invasion. To further support this evidence, future knockdown studies of Claudin‐5 in CPE cells would be needed to determine whether it is a key mediator in HC‐induced barrier strengthening.

Expanding on this, co‐cultures of CPE and breast cancer cells reveal Claudin‐5 protein levels were disrupted relative to control CPE culture at the translational but not the transcriptional level in the presence of HC. Therefore, we speculate that Claudin‐5 downregulation was orchestrated at the post‐transcriptional level. This pattern deviates when observing ZO‐1 levels, as both transcriptional and translational levels failed to demonstrate a decrease in the presence of HC. These findings suggest breast cancer cells are secreting components into their extracellular fluid to disrupt TJs formation, such as Claudin‐5, and assisting in increased BCSFB leakiness. However, TJ disruption may not be specific to Claudin‐5 and further analysis of other TJ components are required.

Surprisingly, we observe patient‐derived breast‐to‐brain metastasis have increased capacity to traverse the BCSFB even in the presence of HC; while migration of primary breast cancer cells across the BCSFB was decreased. This initially puzzling finding is provided greater clarity when observing migration rates independent of barrier resistance. Our findings demonstrate that HC aids breast‐to‐brain metastases cell migration significantly, but primary breast cancers remains unaffected. Although MDA‐MB‐231, MDA‐MB‐231BR, and BBM 3.1 are all triple negative breast tumors, the latter two have formerly colonized the brain parenchyma and have likely developed the critical mechanisms that have allowed them to traverse the CNS' barriers. This leads us to speculate that MDA‐MB‐231 migration is unaided by HC, yet HC functions to strengthen the BCSFB barrier which thwarts barrier traversal as compared to an absence of HC. Consequently, we postulate that high cortisol levels are the basal conditions for breast‐to‐brain metastases. Validating this assertion would require a comparison of the levels of glucocorticoid receptors across primary breast and secondary breast‐to‐brain tumors. Examining GR expression may hold a key to elucidating breast‐to‐brain metastases evolution.

Another unexpected finding was that HC increased migration rates, independent of barrier resistance, in LuBM5 but not A549 cells. A cortisol dependent migration mechanism may potentially exist in lung‐to‐brain metastases. Our study provides some evidence when observing the migration of LuBM5 cells and A549 across the CNS barriers in the presence of HC. While HC fails to significantly curtail migration of LuBM5 cells, it impedes A549 cell migration across both the BBB and BCSFB. The relationship between cortisol and lung tumor metastases has yet to be investigated.

We initially theorized that elevated cortisol levels would decrease breast‐to‐brain metastasis by reinforcing the CNS barrier properties. Although HC decreased migration through the BBB, metastatic cell migration through the BCSFB was sharply higher. In conclusion, we demonstrate that cortisol facilitates breast‐to‐brain metastasis by inducing a more invasive tumor phenotype and breaching the cortisol strengthened BCSFB. Acknowledging the common use of cortisol in the clinic to treat cancer patients,^27^ encourages the scientific community to advance mechanistic studies on cortisol and tumor progression.

## CONFLICT OF INTEREST

The authors declare no conflicts of interest.

## AUTHORS' CONTRIBUTIONS

All authors had full access to the data in the study and take responsibility for the integrity of the data and the accuracy of the data analysis. *Conceptualization*, R.A.H., J.N.; *Methodology*, R.A.H., J.N.; *Investigation*, R.A.H., K.D., V.M., B.S., A.J., R.E.; *Formal Analysis*, R.A.H., J.N.; *Writing ‐ Original Draft*, R.A.H., J.N.; *Writing ‐ Review & Editing*, R.A.H., D.D., M.I., J.N.; *Supervision*, J.N.; *Funding Acquisition*, J.N.

## ETHICAL STATEMENT

All patient‐derived cell lines were obtained through approved University of Southern California Institutional Review Board consent protocol.

## Supporting information


**Figure S1**. A. Treatments of various CM cancer cell lines on BBB and BCSFB, B. Comparative analysis of BBB and BCSFB with CMs at 550 nM HC. All experiments were performed in triplicates. Unpaired Student's *t* test (two tailed) was performed for two groups (0 nM HC and 550 nM HC). For multiple group analysis (A and B), one‐way analysis of variance (ANOVA) with Bonferroni tests was used *P < 0.05, **P < 0.01, ***P < 0.001, ****P < 0.0001. ns, not significant.
**Figure S2**. Effect of HC on Transmigration BC of primary breast cancer SKBr3 (A) and brain‐trophic breast cancer MDA‐MB‐231Br (B) across the BBB or BCSFB. The unpaired Student's *t* test (two tailed) was used to measure significance. **P < 0.01, ***P < 0.001, ****P < 0.0001.

## Data Availability

All data needed to evaluate the conclusions in the article are present in the article and/or the supplementary materials.
