# Supplementary material for: Cortisol promotes breast‐to‐brain metastasis through the blood‐cerebrospinal fluid barrier
Source: Cancer Rep (Hoboken). 2021 Feb 26;5(4):e1351. doi: 10.1002/cnr2.1351 (PMC9124512; doi:10.1002/cnr2.1351)
Supplement: Supplementary file 1 — Figure S1. A. Treatments of various CM cancer cell lines on BBB and BCSFB, B. Comparative analysis of BBB and BCSFB with CMs at 550 nM HC. All experiments were performed in triplicates. Unpaired Student's t test (two tailed) was performed for two groups (0 nM HC and 550 nM HC). For multiple group analysis (A and B), one‐way analysis of variance (ANOVA) with Bonferroni tests was used *P < 0.05, **P < 0.01, ***P < 0.001, ****P < 0.0001. ns, not significant. Figure S2. Effect of HC on Transmigration BC of primary breast cancer SKBr3 (A) and brain‐trophic breast cancer MDA‐MB‐231Br (B) across the BBB or BCSFB. The unpaired Student's t test (two tailed) was used to measure significance. **P < 0.01, ***P < 0.001, ****P < 0.0001. [file CNR2-5-e1351-s001.docx]

**Supporting Information**

Treatments of various CM cancer cell lines on BBB and BCSFB and comparative analysis of BBB and BCSFB with CMs at 550 nM HC are shown in **Supporting information (SI) Figure 1.** All experiments were performed in triplicates.


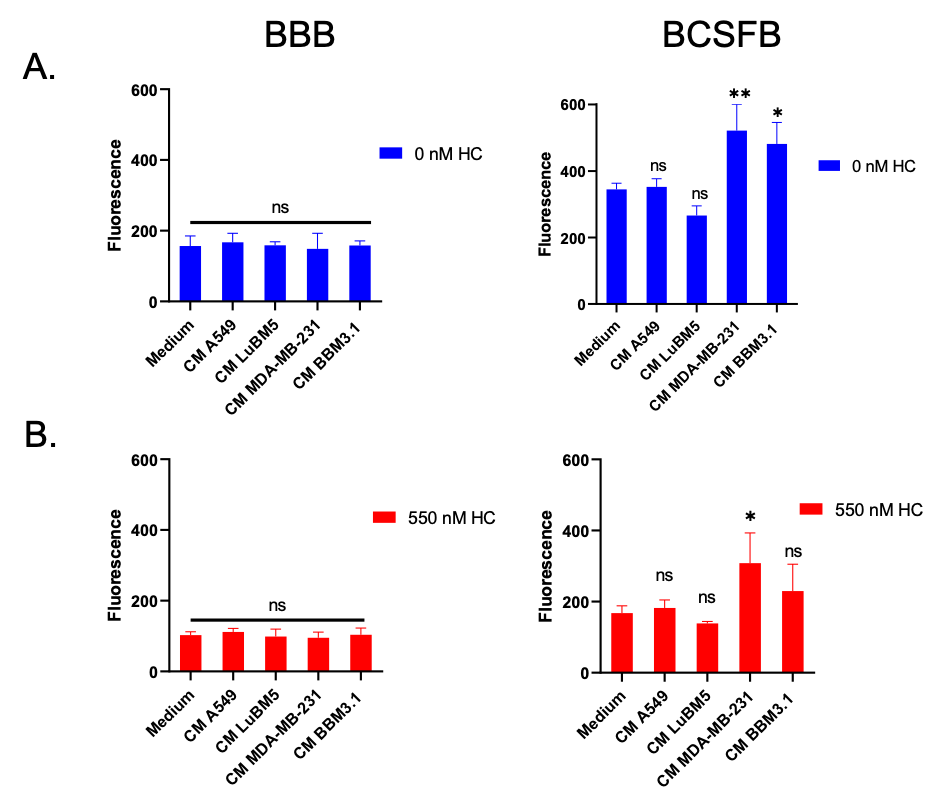


**A**

**B**

**BBB**

**BCSFB**

***SI Figure 1. A.*** Treatments of various CM cancer cell lines on BBB and BCSFB, ***B.*** Comparative analysis of BBB and BCSFB with CMs at 550 nM HC. All experiments were performed in triplicates. Unpaired Student’s *t* test (two tailed) was performed for two groups (0 nM HC and 550 nM HC). For multiple group analysis (A and B), one-way analysis of variance (ANOVA) with Bonferroni tests was used *P < 0.05, **P < 0.01, ***P < 0.001, ****P<0.0001. ns, not significant.

**A**

**B**

***SI Figure 2.*** Effect of HC on Transmigration BC of primary breast cancer SKBr3 (**A)** and brain-trophic breast cancer MDA-MB-231Br **(B)** across the BBB or BCSFB. The unpaired Student’s *t* test (two tailed) was used to measure significance. **P < 0.01, ***P < 0.001, ****P<0.0001
